# Supplementary material for: Unexpectedly efficient ion desorption of graphene-based materials
Source: Nat Commun. 2022 Nov 25;13:7247. doi: 10.1038/s41467-022-35077-9 (PMC9700706; doi:10.1038/s41467-022-35077-9)
Supplement: Supplementary file 1 — Supplementary Information [file 41467_2022_35077_MOESM1_ESM.pdf]

Supplementary Information

Unexpectedly efficient ion desorption of graphene-based materials

Xinming Xia[1,2,3,#], Feng Zhou[4,#], Jing Xu[2,#], Zhongteng Wang[2], Jian Lan[2], Yan Fan[2], Zhikun Wang[2], Wei Liu[2], Junlang Chen[2], Shangshen Feng[2], Yusong Tu[3], Yizhou Yang[5,*], Liang Chen[1,*] and Haiping Fang[5,6,*]

[1]*School of Physical Science and Technology, Ningbo University, Ningbo 315211, China.*

[2]*Department of Optical Engineering, Zhejiang Prov Key Lab Carbon Cycling Forest Ecosy, College of Environmental and Resource Sciences, Zhejiang A&F University, Hangzhou 311300, China.*

[3]*School of Physical Science and Technology & Microelectronics Industry Research Institute, Yangzhou University, Jiangsu 225009, China.*

[4]*Radiation Monitoring Technical Center of Ministry of Environmental Protection, State Environmental Protection Key Laboratory of Radiation monitoring, Key Laboratory of Radiation Monitoring of Zhejiang Province, Hangzhou 310012, China.*

[5]*Department of Physics, East China University of Science and Technology, Shanghai 200237, China.*

[6]*Wenzhou Institute, University of Chinese Academy of Sciences, Wenzhou, Zhejiang 325000, China.*

[#]These authors contributed equally to this work.

*Corresponding author. E-mail: fanghaiping@ecust.edu.cn (H.-P. F.); liangchen@zafu.edu.cn (L. C.); and yangyizhou@ecust.edu.cn (Y.-Z. Y.)

Contents

Supplementary Note 1: Materials and methods 2
Preparation of GO 2
Preparation of M-GO 2
Characterization methods 2
Adsorption and desorption kinetics of Co^{2+}, Mn^{2+}, and Sr^{2+} 2
Adsorption equilibrium of Co^{2+}, Mn^{2+}, Sr^{2+}, and Al^{3+} 3
Effect of Al^{3+} concentration on desorption of Co^{2+}, Mn^{2+}, and Sr^{2+} 3
Adsorption and desorption of radioactive ^{60}Co for enrichment 4
Al^{3+} ions desorption and reusability of M-GO for Co^{2+} adsorption 4
Supplementary Note 2: Characterizations 5
Supplementary Note 3: Equilibrium adsorption capacities of M-GO for Co^{2+}, Mn^{2+}, Sr^{2+}, and Al^{3+} 6
Supplementary Note 4: Ion adsorption and desorption of M-GO for Cu^{2+} and Cd^{2+} 7
Supplementary Note 5: Comparison of the equilibrium rate constant (*k*) of the pseudo-second-order rate on different adsorbents in solutions for multivalent metal ions in the literature 9
Supplementary Note 6: Adsorption and desorption kinetics of the mixed Co^{2+}, Mn^{2+}, and Sr^{2+} salt solution 10
Supplementary Note 7: High-resolution TEM images and magnetic properties of M-GO after each cycle. 10
Supplementary Note 8: Theoretical computations for ions on graphene 11
Supplementary Note 9: Ultraviolet (UV) absorption spectra 11
Supplementary References 12

Supplementary Note 1: Materials and methods

Preparation of GO

Graphene oxide (GO) was prepared from natural graphite powder using a modified Hummers method[1]. Graphite powders was concentrated in H_2SO_4 containing $K_2S_2O_8$ and P_2O_5, and stirred continuously for several hours. Then the mixture was washed and filtered in deionized (DI) water. After vacuum drying, pre-oxidized graphite was obtained. Pre-oxidized graphite was oxidized in concentrated H_2SO_4 and $KMnO_4$, diluted with DI water and further oxidized with H_2O_2 (30%). Then, the product was centrifuged and washed using HCl aqueous solution and DI water. Finally, the GO was obtained and placed in water for further use.

Preparation of M-GO

Magnetite-graphene oxide (M-GO) was prepared through chemical co-precipitation of magnetic iron oxide nanoparticles by coating GO with Fe^{3+} and Fe^{2+} under alkaline conditions[2,3]. Under 353 K and N_2 protection, an acidic mixed aqueous solution of 0.8 g $FeCl_2 \cdot 4H_2O$ and 2.2 g $FeCl_3 \cdot 6H_2O$ was slowly added into the solution containing 0.5 g GO. At 358 K, ammonia solution was quickly added to adjust pH to about 12 and the mixture was stirred vigorously for 60 min. Then, the suspension was cooled to room temperature. By magnetic separation, the solid phase was washed to neutral with DI water, after which it was washed with DI water and ethanol. Finally, M-GO was obtained and placed in water for further use.

Characterization methods

The GO and M-GO were characterized using Raman spectroscopy, XRD, and XPS. The magnetic properties of M-GO were determined using VSM. The morphology of M-GO was characterized using TEM. The concentrations of the ions (Mn^{2+}, Sr^{2+}, Co^{2+}, and Al^{3+}) were determined using Inductively coupled plasma optical emission spectrometer (ICP-OES). The detailed parameters for all instrument characterization are as follows: Raman spectroscopy (horiba evolution), XRD (Panalytical X' Pert Powder), XPS (Thermo Scientific K-Alpha), VSM (Quantum Design PPMS DynaCool), TEM (JEOL JEM 2100), and ICP-OES (Thermo Fisher Scientific iCAP7400).

Adsorption and desorption kinetics of Co^{2+}, Mn^{2+}, and Sr^{2+}

In the adsorption and desorption kinetics study of Co^{2+}, Mn^{2+}, and Sr^{2+}, the prepared M-GO (200 mg) was added to 200 mL solutions of 10 mg/L Co^{2+}, Mn^{2+}, and Sr^{2+}, which were prepared using $CoCl_2 \cdot 6H_2O$, $MnCl_2 \cdot 4H_2O$, and $SrCl_2 \cdot 6H_2O$,

respectively. Subsequently, these mixtures were stirred at 298 K for 125 min. A negligible volume (400 μL) of highly concentrated Al^{3+} solutions (prepared with $AlCl_3 \cdot 6H_2O$) was subsequently added such that the concentration of Al^{3+} in the mixtures was 10 mg/L. The mixtures were then by stirred at 298 K for another 125 min. The volume ratio of Al^{3+} solution to the mixed solution is 1:500. At designated time intervals ranging from 0 to 250 min, 5 mL of the solution was taken at each time interval for filtration separation and the measurements of residual ion concentration. The adsorption capacity (q_t) at time t was calculated according to equation (1):

$$q_t = \frac{(C_0 - C_t)V}{m} \tag{1}$$

where C_0 and C_t (mg/L) are the concentrations of Co^{2+}, Mn^{2+}, Sr^{2+}, or Al^{3+} in the mixed solution at initial and time t, respectively, V (L) is the total volume of the solution, and m (g) is the solid mass of the adsorbent in the aqueous mixture.

In addition, desorption selectivity upon addition of Al^{3+} ions desorption was analyzed. In the adsorption and desorption kinetics study of the mixed solution (Co^{2+}, Mn^{2+}, and Sr^{2+}), the above single component solution was replaced by the mixed solution (10 mg/L Co^{2+}, 10 mg/L Mn^{2+}, and 10 mg/L Sr^{2+}) referring to the above operation method. Then, experiments were carried out according to the above kinetic studies. The adsorption capacity at time t was calculated according to equation (1).

Adsorption equilibrium of Co^{2+}, Mn^{2+}, Sr^{2+}, and Al^{3+}

In the adsorption equilibrium study of Co^{2+}, Mn^{2+}, Sr^{2+}, and Al^{3+}, the prepared M-GO (30 mg) was added to 30 mL solutions of 10 mg/L Co^{2+}, Mn^{2+}, Sr^{2+}, and Al^{3+}. These mixtures were shaken at 160 rpm for 1 h to reach adsorption equilibrium and thereafter separated through magnetic separation and filtration. The ions in the filtrates were determined using ICP-OES. The equilibrium adsorption capacity (q_e, mg/g) of Co^{2+}, Mn^{2+}, Sr^{2+}, and Al^{3+} were calculated from the concentration of initial and equilibrium solutions according to the following equations (2):

$$q_e = \frac{(C_0 - C_e)V}{m} \tag{2}$$

where C_0 and C_e (mg/L) are the initial and equilibrium concentrations of Co^{2+}, Mn^{2+}, Sr^{2+}, or Al^{3+} in the mixed solution during adsorption, respectively, V (L) is the total volume of the solution, and m (g) is the solid mass of the adsorbent in the mixture.

Effect of Al^{3+} concentration on desorption of Co^{2+}, Mn^{2+}, and Sr^{2+}

The effect of Al^{3+} concentration on desorption of Co^{2+}, Mn^{2+}, and Sr^{2+} were studied.

The 30 mL mixture solutions containing 30 mg M-GO and 10 mg/L Co^{2+}, Mn^{2+}, and Sr^{2+} were shaken at 160 rpm and 298 K for 1 h, respectively, which was a continuation of the adsorption experiments. A negligible volume (60 μL) of Al^{3+} solution was thereafter added such that the concentration of Al^{3+} in each ion mixture ranged from 2 to 10 mg/L. The volume ratio of Al^{3+} solution to the mixed solution is 1:500. After shaking for another 1 h, the mixtures were separated through magnetic separation and filtration, and the ions in the filtrates were determined using ICP-OES. The equilibrium adsorption capacity of Al^{3+} was calculated according to equation (2) during desorption. Meanwhile, desorption rate (*D* %) of Co^{2+}, Mn^{2+}, and Sr^{2+} were calculated according to equation (3):

$$D\ (\%) = \frac{C_e' - C_e}{C_0 - C_e} \times 100\% \tag{3}$$

where C_0 and C_e (mg/L) are the initial and equilibrium concentrations of Co^{2+}, Mn^{2+}, Sr^{2+}, or Al^{3+} in the mixed solution during adsorption, respectively. C_e' (mg/L) is the equilibrium concentration of Co^{2+}, Mn^{2+}, Sr^{2+}, or Al^{3+} during desorption.

Adsorption and desorption of radioactive ^{60}Co for enrichment

The enrichment multiple (*n*) was calculated from the activity concentrations of the initial and desorbed solutions according to equation (4):

$$n = \frac{C_3}{C_1} \tag{4}$$

where C_1 and C_3 (Bq/L) are the activity concentrations of ^{60}Co of initial and desorbed solution, respectively.

Al^{3+} ions desorption and reusability of M-GO for Co^{2+} adsorption

The 30 mL mixture solutions containing 30 mg M-GO and 10 mg/L Al^{3+} were shaken at 298 K for 5 min to reach adsorption equilibrium, which was a continuation of the above Al^{3+} adsorption experiments. 75 μL $NH_3 \cdot H_2O$ (25~28%) was added to the 30 mL mixture solutions to adjust the pH to 10, and then the mixtures were separated through magnetic separation and filtration. The separated M-GO was desorbed again with 30 mL DI water (containing 75 μL $NH_3 \cdot H_2O$). Finally, the recycled M-GO was obtained after two desorption steps. The concentration of Al^{3+} in the filtrates were determined using ICP-OES. The q_e and desorption rate of Al^{3+} were calculated according to equation (2) and (3).

In the re-adsorption on recycled M-GO for Co^{2+}, the 10 mg/L Co^{2+} re-adsorption by recycled 30 mg M-GO in the 30 ml mixture solution was carried out, and recycled

through Co^{2+} desorption, Al^{3+} desorption, and Co^{2+} adsorption. The desorption process of Al^{3+} on M-GO was described above, and other experimental operations can refer to the adsorption and desorption of Co^{2+}. Finally, the q_e was calculated according to equation (2).

Supplementary Note 2: Characterizations

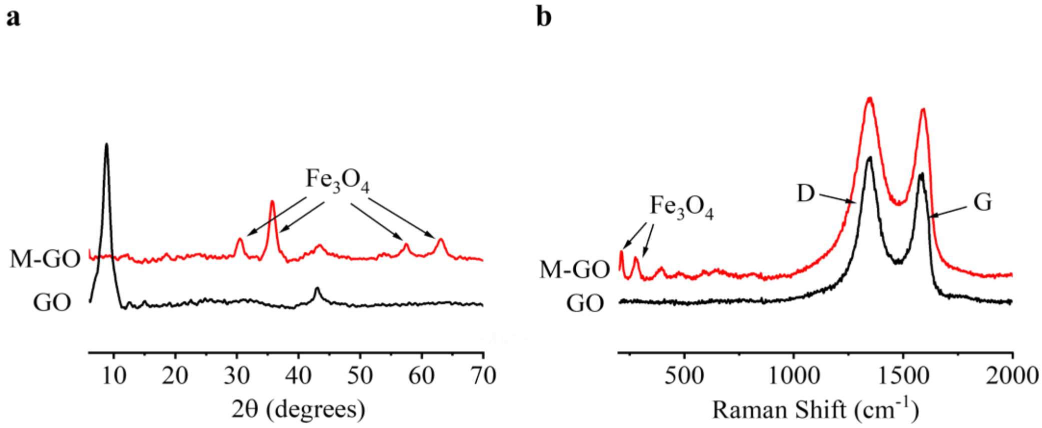

Supplementary Fig. 1. a XRD pattern of GO and M-GO, and **b** Raman spectra (λ=532 nm) of GO and M-GO.

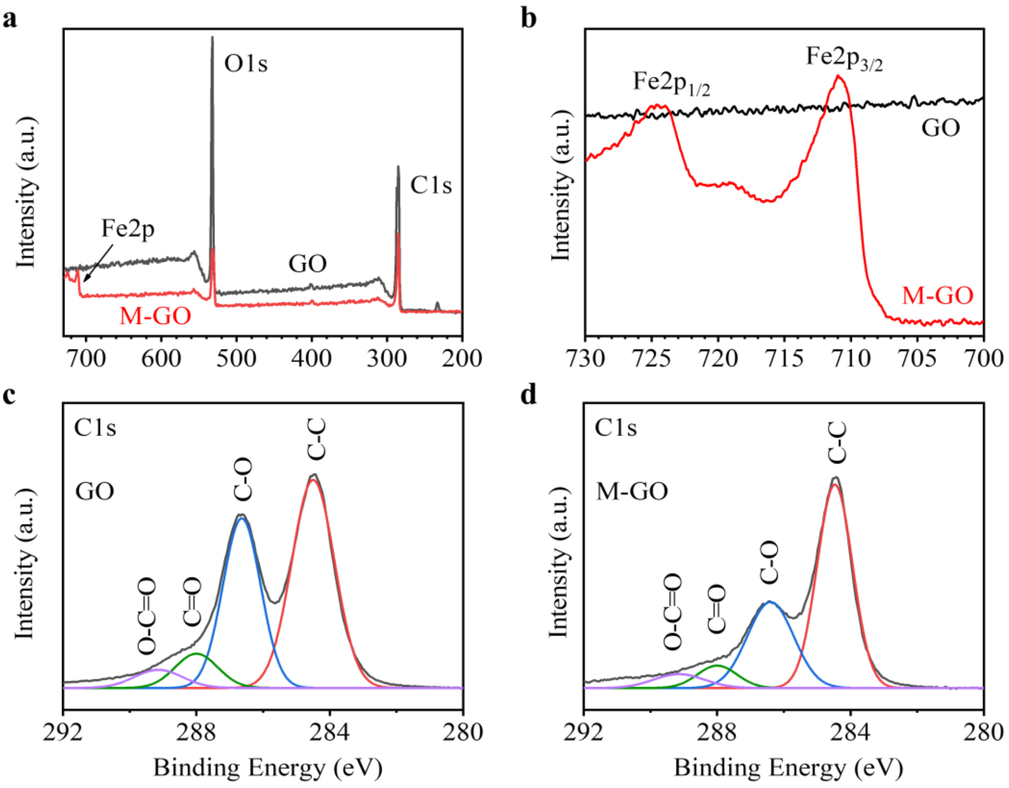

Supplementary Fig. 2 | XPS spectra: **a** wide scan of GO and M-GO, **b** Fe2p spectra of GO and M-GO, **c** C1s spectra of GO, and **d** C1s spectra of M-GO.

Supplementary Note 3: Equilibrium adsorption capacities of M-GO for Co^{2+}, Mn^{2+}, Sr^{2+}, and Al^{3+}

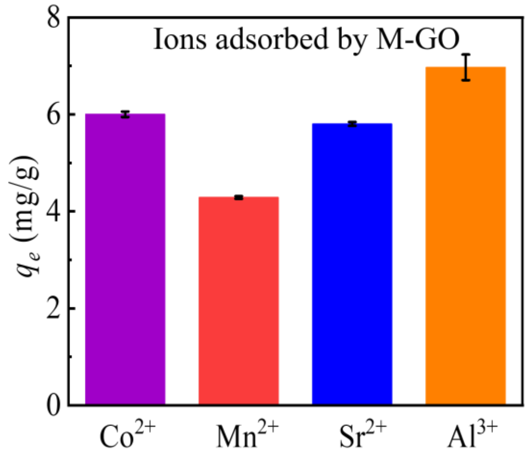

Supplementary Fig. 3 | Equilibrium adsorption capacities of M-GO for 10 mg/L Co^{2+}, Mn^{2+}, Sr^{2+}, and Al^{3+}, respectively. Error bars indicate the standard deviation from three different samples.

The experimental operation of equilibrium adsorption capacities of M-GO for Co^{2+}, Mn^{2+}, Sr^{2+}, and Al^{3+} can be found in Supplementary Note 1. As shown in Supplementary Fig. 3, the equilibrium adsorption capacities of M-GO for Co^{2+}, Mn^{2+}, Sr^{2+}, and Al^{3+} solutions were 6.0 ± 0.1, 4.2 ± 0.1, 5.8 ± 0.1, and 7.0 ± 0.3 mg/g, respectively, which is consistent with those of previous reports[2–4]. This demonstrates the efficient ion removal effect of M-GO, indicating that the M-GO adsorbed ions can be conveniently separated from the aqueous solution through magnetic solid/liquid separation, consistent with previous reports[5].

Supplementary Note 4: Ion adsorption and desorption of M-GO for Cu^{2+} and Cd^{2+}

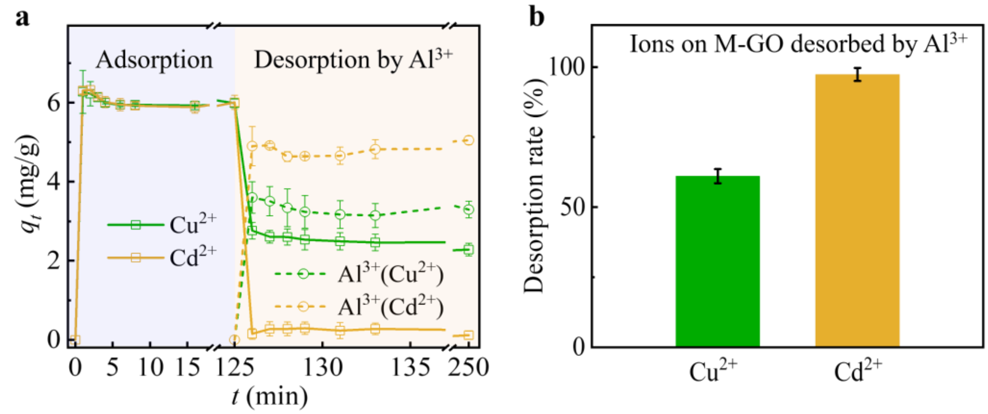

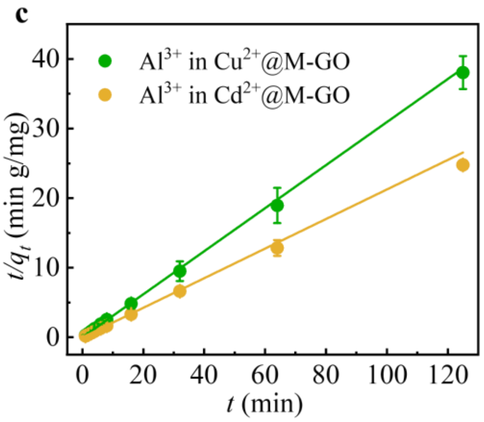

Adsorption kinetic parameters of Al^{3+} by pseudo-second-order rate models.

Target ions	$q_{e\ exp}$ (mg/g)	k (g/mg min^{-1})	$q_{e\ cal}$ (mg/g)	R^2
Al^{3+} in Cu^{2+}@M-GO	3.4	130.2	3.2	0.9989
Al^{3+} in Cd^{2+}@M-GO	4.8	60.4	4.7	0.9994

Supplementary Fig. 4 | Ion adsorption and desorption of M-GO for Cu^{2+} and Cd^{2+}. a Adsorption kinetics of 10 mg/L Cu^{2+} and Cd^{2+}, as well as adsorption kinetics of 10 mg/L Al^{3+} added to the salt solutions (Cu^{2+} and Cd^{2+}) at 298 K, respectively. q_t denotes the adsorption capacity of M-GO with time. Light purple and light orange are highlighted to distinguish between adsorption and desorption. **b** Desorption rate of Cu^{2+} and Cd^{2+} from M-GO by the subsequent addition of Al^{3+}. **c** Adsorption kinetic parameters of Al^{3+} during the ion desorption of Cu^{2+} and Cd^{2+} by a pseudo-second-order rate model. Error bars indicate the standard deviation from three different samples.

The ion adsorption and desorption kinetics experiments of M-GO for Cu^{2+} and Cd^{2+} were further carried out, and the experimental procedures were consistent with those of Co^{2+}, Mn^{2+}, and Sr^{2+}, refer to Supplementary Note 1. 10 mg/L Cu^{2+} and Mn^{2+} solutions were prepared with $CuCl_2 \cdot 2H_2O$ and $CdCl_2 \cdot 2.5H_2O$, respectively.

As shown in Supplementary Fig. 4a, rapid ion adsorption of Cu^{2+} and Cd^{2+} adsorbed by M-GO occurred within 1 min after adding ions, and the equilibrium adsorption capacities of M-GO are 6.0 ± 0.2 and 5.9 ± 0.2 mg/g for Cu^{2+} and Cd^{2+}, respectively. Then a rapid desorption of Cu^{2+} and Cd^{2+} ions was observed when 10 mg/L Al^{3+} ions were added at 125 min. The corresponding desorption rate were 61.1 ±

2.5% and 97.3 ± 2.3% for Cu^{2+} and Cd^{2+}, respectively (Supplementary Fig. 4b). We note that the adsorption energy of Cu^{2+}@G is much higher than those of other divalent ions (Co^{2+}, Mn^{2+}, Sr^{2+}, and Cd^{2+})[6,7], resulting in the moderate desorption of Cu^{2+} under the same conditions. However, a satisfied desorption of Cu^{2+} can be obtained by successive addition of Al^{3+} ions, for example, the desorption rate of Cu^{2+} can reach 82.9 ± 0.2% after the second desorption cycle, yielding the desorption performance still superior to that of conventional desorption methods.

The calculated adsorption capacities ($q_{e\ cal}$) listed in Supplementary Fig. 4c, are consistent with the corresponding experimental values ($q_{e\ exp}$), indicating that the kinetic adsorption can be well described by the pseudo-second-order rate equation. The k values of Al^{3+} during the desorption of the Cu^{2+} and Cd^{2+} ions are 130.2 and 60.4 g/mg min^{-1}, respectively, which are much higher than the equilibrium rate constants of other types of adsorbents (Supplementary Table 1). We noted that the k of Al^{3+} in the desorption process of Cu^{2+} is higher than that in other divalent ions (Co^{2+}, Mn^{2+}, Sr^{2+}, and Cd^{2+}), which we attribute to the less amount of Cu^{2+} desorption, allowing a shorter time to reach desorption equilibrium, thus resulting in a faster adsorption kinetic parameter. In all, these results indicate that the rapid desorption of the Cu^{2+} and Cd^{2+} ions also can be achieved by our method, indicating a wide application range of the method in this work.

Supplementary Note 5: Comparison of the equilibrium rate constant (*k*) of the pseudo-second-order rate on different adsorbents in solutions for multivalent metal ions in the literature

Supplementary Table 1 | Comparison of the *k* of the pseudo-second-order rate on different adsorbents in solutions for multivalent metal ions in the literature.

Adsorbents	Target ions	k (g/mg min^{-1})	References
ZIF-8	Cu^{2+}	2.81×10^{-1}	Ref.[8]
ZF-NPS	Cd^{2+}	1.65×10^{-1}	Ref.[9]
BC@MnO_2	Cu^{2+}	4.60×10^{-3}	Ref.[10]
	Cu^{2+}	5.58×10^{-3}	Ref.[11]
MoS_4-LDH	Pb^{2+}	2.71×10^{-1}	Ref.[11]
	Hg^{2+}	3.62×10^{-1}	Ref.[11]
Chitosan	Al^{3+}	1.68×10^{-3}	Ref.[12]
	Cu^{2+}	1.55×10^{-2}	Ref.[13]
GO membranes	Cd^{2+}	1.79×10^{-2}	Ref.[13]
	Ni^{2+}	1.05×10^{-2}	Ref.[13]
	Co^{2+}	9.40×10^{-3}	Ref.[14]
GO/cellulose membranes	Zn^{2+}	9.70×10^{-3}	Ref.[14]
	Pb^{2+}	5.50×10^{-3}	Ref.[14]
VA-PG	UO_2^{2+}	9.10×10^{-7}	Ref.[15]
GO-$MnFe_2O_4$	Pb^{2+}	9.50×10^{-1}	Ref.[16]
PANI/GO	Sr^{2+}	9.50×10^{-3}	Ref.[17]
MGO-PP	UO_2^{2+}	1.90×10^{-3}	Ref.[18]
M-RGO	As^{3+}	5.00×10^{-2}	Ref.[19]
	Co^{2+}	1.86×10^{-2}	Ref.[2]
	Cu^{2+}	1.10×10^{-3}	Ref.[20]
	Fe^{2+}	47.2	Ref.[3]
	Mn^{2+}	43.6	Ref.[3]
M-GO	Al^{3+} (in Co^{2+} solution)	55.3	this work
	Al^{3+} (in Mn^{2+} solution)	51.3	this work
	Al^{3+} (in Sr^{2+} solution)	41.8	this work
	Al^{3+} (in Cu^{2+} solution)	130.2	this work
	Al^{3+} (in Cd^{2+} solution)	60.4	this work

Supplementary Note 6: Adsorption and desorption kinetics of the mixed Co^{2+}, Mn^{2+}, and Sr^{2+} salt solution

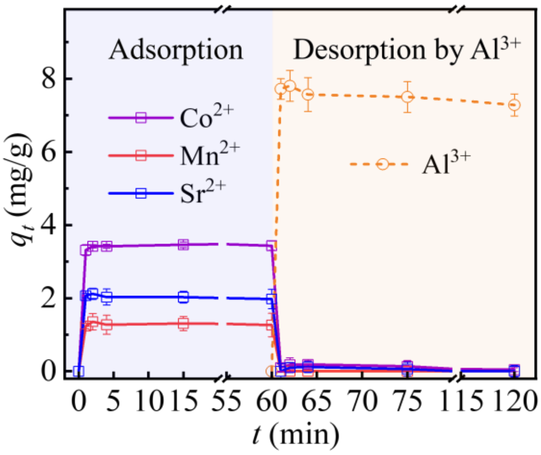

Supplementary Fig. 5 | Adsorption kinetics of the mixed salt solution (10 mg/L Co^{2+}, 10 mg/L Mn^{2+}, and 10 mg/L Sr^{2+}) by M-GO, as well as adsorption kinetics of 10 mg/L Al^{3+} added into the mixed solutions at 298 K. Error bars indicate the standard deviation from three different samples.

Supplementary Note 7: High-resolution TEM images and magnetic properties of M-GO after each cycle.

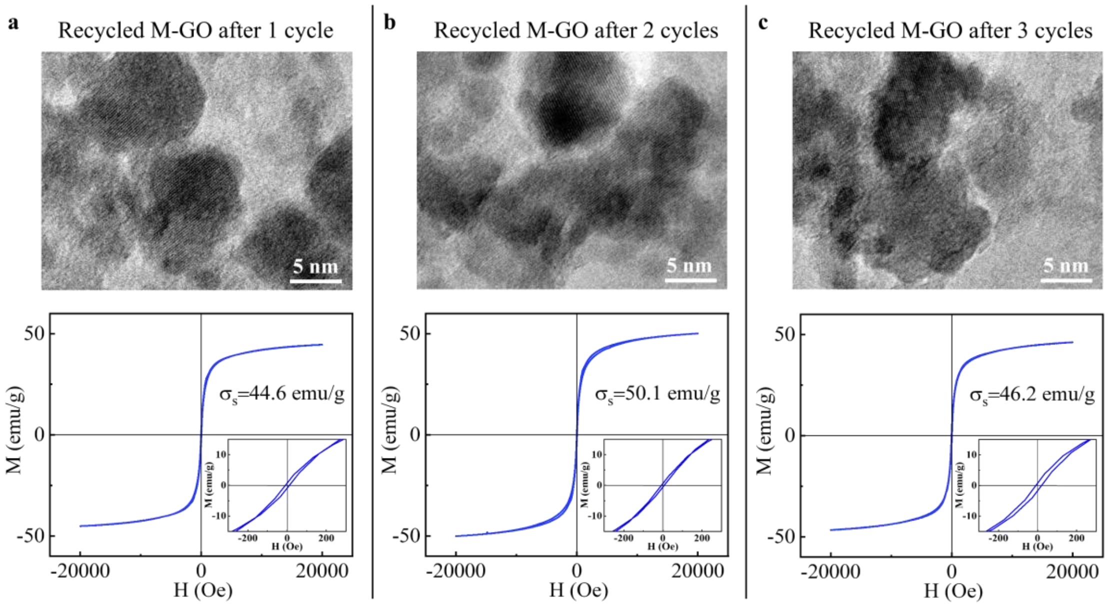

Supplementary Fig. 6 | High-resolution TEM images and magnetic properties of M-GO after each cycle. The high-resolution TEM images (top) and magnetization curve at room temperature (down) for the M-GO after **a** 1 cycle, **b** 2 cycles, and **c** 3 cycles.

Supplementary Note 8: Theoretical computations for ions on graphene

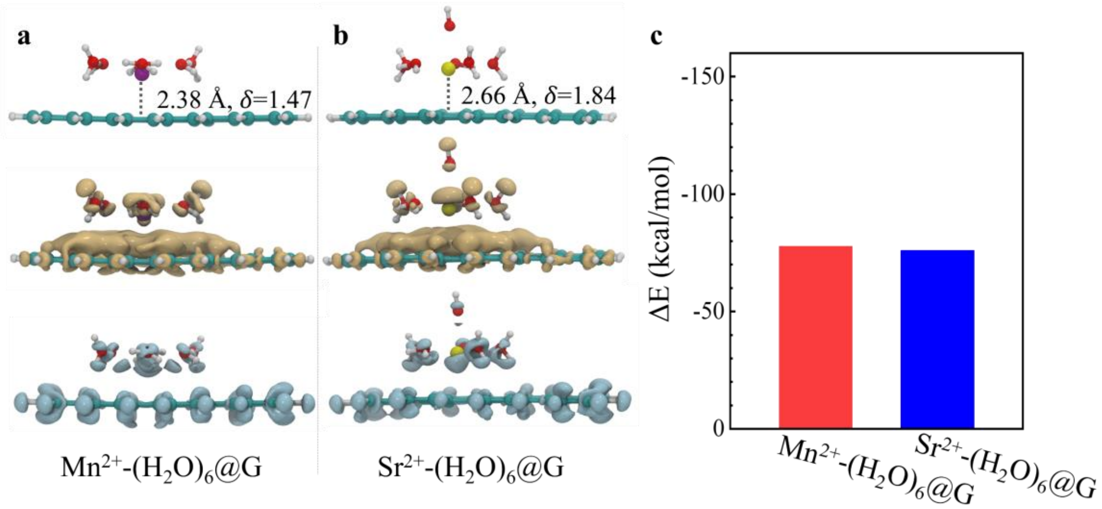

Supplementary Fig. 7 | Theoretical computations for ions on graphene. The most stable optimized geometries and electron density differences of X@G complexes: **a** X is Mn^{2+}-$(H_2O)_6$, **b** X is Sr^{2+}-$(H_2O)_6$. Spheres in green, white, and red represent carbon, hydrogen, and oxygen atoms, respectively. purple and yellow spheres represent Mn^{2+} and Sr^{2+}, respectively. Adsorption distances (in Å) and partial charges of cation δ (in atomic units) are listed. The increased and decreased electron densities (from −0.1 to 0.1) are in khaki and grey, respectively. **c** The calculated adsorption energies of X@G at the level of M06-2X/Def2-SVP.

Supplementary Note 9: Ultraviolet (UV) absorption spectra

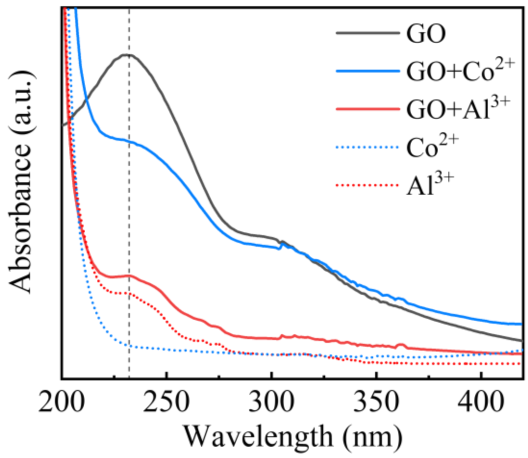

Supplementary Fig. 8 | UV absorption spectra. UV absorption spectra of GO suspension (50 mg/L) mixed in a 1:1 ratio with 0.4 M $CoCl_2$ and $AlCl_3$ solutions (blue and red solid line) together with GO suspension in pure water (black solid line), and pure 0.4 M $CoCl_2$ and $AlCl_3$ salt solution (blue and red dashed line).

The UV absorption spectra of GO and GO in $CoCl_2$ and $AlCl_3$ solution are showed in Supplementary Fig. 8. The UV spectrum of GO at ~230 nm, which is assigned to a conjugate double bond of the aromatic group that easily generated π-π* transition[21–23]. Compared with the UV intensity of GO in $CoCl_2$ solution, the intensity of GO in $AlCl_3$ solution markedly decreased, indicating that the conjugate double bonds of the aromatic group in GO are greatly affected in $AlCl_3$ solution.

Supplementary References

1. Hummers, *et al.* Preparation of Graphitic Oxide. *J. Am. Chem. Soc.* **80**, 1339 (1958).
2. Liu, M., Chen, C., Hu, J., Wu, X. & Wang, X. Synthesis of Magnetite/Graphene Oxide Composite and Application for Cobalt (II) Removal. *J. Phys. Chem. C* **115**, 25234–25240 (2011).
3. Yan, H. *et al.* Rapid Removal and Separation of Iron (II) and Manganese (II) from Micropolluted Water Using Magnetic Graphene Oxide. *ACS Appl. Mater. Interfaces* **6**, 9871–9880 (2014).
4. Minitha, C. R. *et al.* Magnetite Nanoparticle Decorated Reduced Graphene Oxide Composite as an Efficient and Recoverable Adsorbent for the Removal of Cesium and Strontium Ions. *Ind. Eng. Chem. Res.* **57**, 1225–1232 (2018).
5. Lingamdinne, L. P., Koduru, J. R. & Karri, R. R. A comprehensive review of applications of magnetic graphene oxide based nanocomposites for sustainable water purification. *J. Environ. Manage.* **231**, 622–634 (2019).
6. Shi, G. *et al.* Ion Enrichment on the Hydrophobic Carbon-based Surface in Aqueous Salt Solutions due to Cation-π Interactions. *Sci. Rep.* **3**, 3436 (2013).
7. Li, J. et al. "On-off-on" fluorescence switch of graphene quantum dots: A cationic control strategy. *Appl. Surf. Sci.* **546**, 149110 (2021).
8. Latrach, Z., Moumen, E., Kounbach, S. & El Hankari, S. Mixed-Ligand Strategy for the Creation of Hierarchical Porous ZIF-8 for Enhanced Adsorption of Copper Ions. *ACS Omega* **7**, 15862–15869 (2022).
9. Zhao, X., Baharinikoo, L., Farahani, M. D., Mahdizadeh, B. & Farizhandi, A. A. K. Experimental modelling studies on the removal of dyes and heavy metal ions using $ZnFe_2O_4$ nanoparticles. *Sci. Rep.* **12**, 5987 (2022).
10. Zhang, H. et al. Enhanced removal of heavy metal ions from aqueous solution using manganese dioxide-loaded biochar: Behavior and mechanism. *Sci. Rep.* **10**, 1–13 (2020).
11. Ma, L. et al. Highly Selective and Efficient Removal of Heavy Metals by Layered Double Hydroxide Intercalated with the MoS_4^{2-} Ion. *J. Am. Chem. Soc.* **138**, 2858–

2866 (2016).
12. Septhum, C., Rattanaphani, S., Bremner, J. B. & Rattanaphani, V. An adsorption study of Al(III) ions onto chitosan. *J. Hazard. Mater.* **148**, 185–191 (2007).
13. Tan, P. et al. Adsorption of Cu^{2+}, Cd^{2+} and Ni^{2+} from aqueous single metal solutions on graphene oxide membranes. *J. Hazard. Mater.* **297**, 251–260 (2015).
14. Sitko, R., Musielak, M., Zawisza, B., Talik, E. & Gagor, A. Graphene oxide/cellulose membranes in adsorption of divalent metal ions. *RSC Adv.* **6**, 96595–96605 (2016).
15. Liu, T. et al. Vertically Aligned Polyamidoxime/Graphene Oxide Hybrid Sheets' Membrane for Ultrafast and Selective Extraction of Uranium from Seawater. *Adv. Funct. Mater.* **32**, 2111049 (2022).
16. Kumar, S. et al. Graphene Oxide–$MnFe_2O_4$ Magnetic Nanohybrids for Efficient Removal of Lead and Arsenic from Water. *ACS Appl. Mater. Interfaces* **6**, 17426–17436 (2014).
17. Hu, B. et al. Decontamination of Sr(II) on Magnetic Polyaniline/Graphene Oxide Composites: Evidence from Experimental, Spectroscopic, and Modeling Investigation. *ACS Sustainable Chem. Eng.* **5**, 6924–6931 (2017).
18. Dai, Z., Sun, Y., Zhang, H., Ding, D. & Li, L. Highly Efficient Removal of Uranium(VI) from Wastewater by Polyamidoxime/Polyethyleneimine Magnetic Graphene Oxide. *J. Chem. Eng. Data* **64**, 5797–5805 (2019).
19. Chandra, V. et al. Water-Dispersible Magnetite-Reduced Graphene Oxide Composites for Arsenic Removal. *ACS Nano* **4**, 3979–3986 (2010).
20. Li, J. et al. Removal of Cu(II) and Fulvic Acid by Graphene Oxide Nanosheets Decorated with Fe_3O_4 Nanoparticles. *ACS Appl. Mater. Interfaces* **4**, 4991–5000 (2012).
21. Shi, G. et al. Unexpectedly Enhanced Solubility of Aromatic Amino Acids and Peptides in an Aqueous Solution of Divalent Transition-Metal Cations. *Phys. Rev. Lett.* **117**, 238102 (2016).
22. Chuang, C. H. & Chen, Y. T. Raman scattering of L-tryptophan enhanced by surface plasmon of silver nanoparticles: vibrational assignment and structural determination. *J. Raman Spectrosc.* **40**, 150-156 (2009).
23. Yorita, H. et al. Evidence for the cation-π interaction between Cu^{2+} and tryptophan. *J. Am. Chem. Soc.* **130**, 15266-15267 (2008).
